# Targeting invadopodia to block breast cancer metastasis

**DOI:** 10.18632/oncotarget.301

**Published:** 2011-06-30

**Authors:** Mark A. Eckert, Jing Yang

**Affiliations:** ^1^Department of Pharmacology; ^2^The Molecular Pathology Graduate Program; ^3^Department of Pediatrics

**Keywords:** Twist1, PDGFR, Src, MMP, MT1-MMP, SFK, metastasis, invasion, breast cancer, invadopodia, podosomes

## Abstract

Better understanding the mechanisms underlying the metastatic process is essential to developing novel targeted therapeutics. Recently, invadopodia have been increasingly recognized as important drivers of local invasion in metastasis. Invadopodia are basally-localized, actin-rich structures that concentrate protease activity to areas of the cell in contact with the extracellular matrix. We recently found that the transcription factor Twist1, a central regulator of the epithelialmesenchymal transition (EMT), promotes invadopodia formation via upregulation of platelet-derived growth factor receptor (PDGFR) expression and activity. This finding, combined with other investigations into the mechanisms of invadopodia formation, reveal several novel targets for clinical inhibition of invadopodia. Here, we provide an overview of clinically-relevant targets for intervention in invadopodia, including Src signaling, PDGFR signaling, and metalloprotease activity.

## BREAST CANCER METASTASIS, EMT, AND THE STATE OF THERAPEUTIC INTERVENTIONS

Breast cancer is the most common invasive cancer among women worldwide, with virtually all patients succumbing to not the primary disease, but distant metastases[1, 2]. Metastasis, the spread of cancer cells from the primary tumor to distant organs, is a multistep process in which cancer cells must invade through the extracellular matrix (ECM), intravasate into the bloodstream, survive transport through the circulatory system, and finally extravasate at distant organs[3]. As metastatic breast cancer is largely considered an incurable disease, better understanding the metastatic process and its regulation has the potential to not only identify new prognostic markers but also develop targeted therapeutic regimens.

Recently, aberrant activation of a developmental program termed the epithelial-mesenchymal transition (EMT) has been recognized as an important driver of the metastatic process[4].EMT is a conserved developmental process in which epithelial cells lose E-cadherin-mediated junctions and apical-basal polarity and become motile and invasive [5]. This program is accompanied by expression changes in a host of genes, among which genes associated with epithelial characteristics (E-cadherin and ZO-1) are downregulated while others associated with mesenchymal cells (smooth muscle actin, vimentin, and N-cadherin) are upregulated. A group of transcription factors, including Twist1, Snai1, Snai2, Zeb1, and Zeb2, play key roles in driving EMT during tumor metastasis[6, 7].

Current therapeutic standards for breast cancer involve surgical resection of the tumor supplemented with radiation therapy and chemotherapy[8]. Cytotoxic drugs and hormone-blocking therapeutics are the most often used chemotherapeutics, generally chosen for their effects on cell growth and apoptosis. Generation of new therapeutic agents targeting invasion and metastasis have the potential to improve survival in populations that do not respond well to conventional therapies. Despite the growing evidence linking EMT to metastasis in breast and other cancers, therapeutically targeting EMT may be difficult. Directly inhibiting the transcription factors that drive EMT is currently infeasible, as targeting large binding interfaces is not amenable to small-molecule inhibition[9, 10]. Instead, downstream targets of these transcription factors essential for their role in invasion and metastasis are more realistic targets of therapeutic intervention.

## TWIST1 AND INVADOPODIA

Although the role of EMT in metastasis is gradually becoming clearer, the exact molecular mechanisms underlying how EMT induces local invasion and metastasis are still not well understood[11]. Disruption of epithelial cell-cell contact is necessary for metastasis, but it is not sufficient[12]. We therefore sought to determine what pathways or mechanisms Twist1 induces to drive active local invasion and metastasis. We did not observe significant changes in secreted proteolytic activity in cells overexpressing Twist1, although they gained the ability to invade through Matrigel and metastasize to the lung in a subcutaneous tumor model[7]. We therefore hypothesized that Twist1 induces local invasion and eventual metastasis by inducing the formation of membrane protrusion structures called invadopodia.

Invadopodia are actin-rich protrusions that localize proteolytic activity to areas of the cell in contact with extracellular matrix(ECM)[13-15]. Invadopodia are observed in many invasive cancer cell lines [16]. A wide variety of actin-interacting proteins and scaffolding proteins are involved in invadopodia formation, including cortactin, Tks5, fascin, N-WASP, and Arp2/3[17]. In particular, the actin-bundling protein cortactin and the adaptor proteins Tks4/5appear to play integral roles in invadopodia formation[18, 19]. Both metalloproteases and serine proteases localize to invadopodia, including both secreted (MMP2 and MMP9) and transmembrane proteases (MT1-MMP, ADAM12, FAPα, and DPP-iv)[20]. Src kinase activity and phosphorylation of Tks4 [21], Tks5[18], and cortactin[19]are absolute requirements for invadopodia formation. Upregulation of invadopodia formation by Twist1 would therefore present a novel mechanism by which Twist1 could induce local invasion without changing secreted protease activity[22].

In order to investigate whether Twist1 was necessary for invadopodia, we generated knockdowns of Twist1 in 168FARN and 4T1 cell lines, two mouse mammary carcinoma cell lines that express high levels of Twist1. By staining for markers of invadopodia (colocalization of F-actin with either cortactin or Tks5) we found that knockdown of Twist1 significantly reduced invadopodia formation in both 168FARN and 4T1 cells[23]. Importantly, knockdown of Twist1 also dramatically reduced ECM degradation.Similar analyses in normal mammary epithelial cells overexpressing Twist1 demonstrated that Twist1 was also sufficient to promote invadopodia formation and function. Importantly, Twist1-inducedinvadopodia formation requires both metalloprotease and Src-kinase activities, consistent with their known roles in invadopodia.

## TWIST1 INDUCES INVADOPODIA FORMATION BY UPREGULATING PDGFRα

We were therefore interested in the mechanism by which Twist1 was both necessary and sufficient for invadopodia formation. None of the structural or enzymatic proteins found in invadopodia that we investigated, including Tks5, cortactin,and MT1-MMP, were transcriptionally regulated by Twist1. We did, however, observe a significant upregulation of Src kinase activation upon Twist1 expression. Microarray analysis of genes upregulated by Twist1 revealed that only one family of growth factor receptors upstream of Srcactivation was induced by Twist1: platelet-derived growth factor receptors (PDGFR) α and β. PDGFRα, in particular, was immediately and dramatically upregulated. Importantly, PDGFRα was phosphorylated and activated under normal culture conditions, implying the existence of an autocrine loop upon Twist1 activation.

There are two PDGFRs, PDGFRα and β, which differ primarily in their responsiveness to PDGF ligands[24]. In mammalian systems, PDGFR expression is abundant in mesenchymal and vascular tissues and is particularly involved in angiogenesis[25, 26]. Importantly, PDGFRs are directly upstream of Src kinase activity[27]. Upon stimulation by their ligands, the receptors dimerize and can directly activate Src kinase[28]. PDGFR signaling has previously been implicated as required for metastasis in a TGF-β-induced EMT model, although the mechanism for this inhibition was not clearly understood[29]. Encouragingly, a previous study found that PDGFR activation increased invadopodia formation in vascular smooth muscle cells[30].

Knockdown or inhibition of PDGFRα with a mouse monoclonal blocking antibody significantly reduced Twist1-induced invadopodia formation and function. Chromatin immunoprecipitation and luciferase reporter assays also revealed that PDGFRα was a direct target of Twist1. Activation of PDGFR signaling by Twist1 therefore appeared to be a direct requirement for Twist1-mediated invadopodia formation.

## INVADOPODIA AND PDGFRα ARE NECESSARY FOR METASTASIS AND IMPLICATED IN HUMAN BREAST CANCER

To better understand the role of invadopodia and PDGFRα in metastasis, we utilized a subcutaneous tumor implantation model in which Twist1-expressing human breast tumor cells carrying shRNAs against PDGFRα or Tks5 were injected subcutaneously in nude mice. Knockdown of Tks5, an essential invadopodia component protein with no other known functions, allowed us to test whether invadopodia were required for Twist1-mediated metastasis. Knockdown of PDGFRα was used to determine whether PDGF signaling induced by Twist1 for invadopodia formation was required for metastasis. Although no significant differences in growth rate were observed, both Tks5 and PDGFRα knockdowns dramatically suppressed local invasion, with the primary tumors remaining relatively well-encapsulated in a layer of fibrotic tissue. In contrast, tumors expressing control knockdown constructs invaded through the local ECM, often as single cells. Furthermore, knockdown of both Tks5 and PDGFRα significantly reduced dissemination to the lungs, as measured by quantification of GFP-positive puncta in the lungs.

In microarray data sets of human breast cancer tumor samples, we found a strong correlation between Twist1 and PDGFRα expression, with PDGFRα consistently ranking within the top 1% of genes correlated with Twist1. Furthermore, in a Stage II breast cancer tissue array from the National Cancer Institute coexpression of Twist1 and PDGFRα was significantly associated with patient survival, indicating the importance of this pathway in human breast tumor progression.

Druggable targets regulating invadopodia formation and function

**Figure 1:**
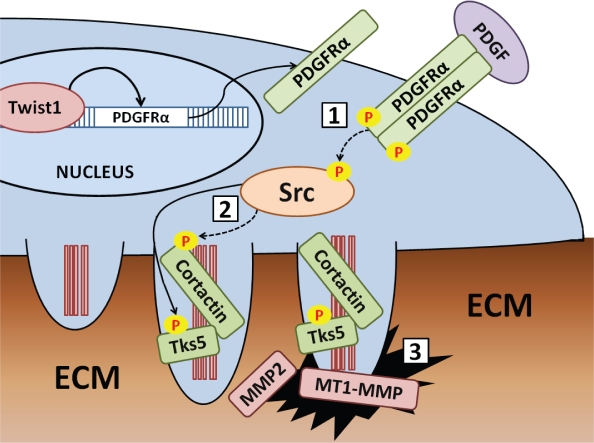
Recent research in our lab revealed that Twist1 directly induces transcription of PDGFRα Upregulation of PDGFRα leads to an increase in Src kinase activity that induces the formation or stabilization of invadopodia by phosphorylation of invadopodia component proteins by Src kinase. Invadopodia formation involves discrete steps in which formation of the F-actin core is an early event, followed by recruitment and phosphorylation of invadopodia component proteins like cortactin and Tks5 before proteases are recruited to the mature invadopodia. Promising targets for chemical inhibition include [1] PDGFR signaling, [2] Src kinase activity, and [3] metalloprotease activity at invadopodia (including MMP2, MMP9, and MT1-MMP).

The connection between Twist1, PDGFR signaling, and invadopodia is exciting as it highlights several new therapeutic targets for targeting local invasion in the metastatic process. Namely, PDGFRα, Src, and metalloproteases localized in invadopodia are appealing, druggable targets for targeting invasion in breast cancer metastasis (see Figure 1). As metastasis may occur via invadopodia-independent mechanisms in patient populations, Twist1 and PDGFRα coexpression may be appealing markers for patient stratification for treatment regimens targeting invadopodia.

### PDGFRα and EGFR

As a direct target of Twist1 tightly associated with survival in human breast cancer patient tissue samples, PDGFRα is an especially appealing target for therapeutic intervention in breast cancer metastasis. The most well-known and studied PDGFR inhibitor is imatinib mesylate (Gleevec, Novartis), which also inhibits Abl and c-Kit tyrosine kinases[31]. Data from clinical trials involving use of imatinib in advanced breast cancers has been discouraging with no clear objective responses[32]. If PDGFR signaling is important for invasion and metastasis, however, improved survival in these patients with late stage disease would be unlikely as the cancer had already widely metastasized. Often severe gastrointestinal side effects of imatinib treatment also severely limited its utility in at least one trial[33]. Another tyrosine kinase inhibitor, Sunitinib (Sutent, Pfizer), targets PDGFRs, VEGFs, Kit, RET, and CSF[33]. Encouragingly, Sunitinib is much better tolerated and has had some effectiveness in preliminary clinical trials of metastatic breast cancer[34]. The promiscuous inhibitory profile of Sunitinib makes it difficult to determine whether its effects on disease outcome are through inhibition of PDGFRs. In light of our discovery, it is important to examine patient tumor samples to determine whether Sunitinib suppresses invadopodia and local invasion. To truly understand the utility of these novel inhibitors in breast cancer, it will be necessary to identify patient populations that will respond best to the therapy. Development of more specific inhibitors that target only PDGFRs, including humanized monoclonal antibodies, may address some of the side effects due to off targeting.

Epidermal growth factor receptor (EGFR) signaling is also known to play an important role in regulation of invadopodia formation. The most characterized role of EGFR signaling in invadopodia is its function upstream of Src activation[35]. HER-2 (human epidermal growth factor receptor 2) status is an important clinical marker for treatment, with about 30% of patients presenting increased levels of HER-2expression[33]. Patients that are HER-2 positive are considered candidates for treatment with HER-2 inhibitors, including trastuzumab (Herceptin, Genentech) and lapatinib (Tykerb/Tyverb GSK)[36]. Recently, new small molecule-based therapeutics targeting EGFR, including erlotinib (Tarceva, OSI Pharmaceuticals), have proven useful in other cancers with upregulation of EGFR signaling[37]. OSI Pharmaceuticals investigated the properties of cancer cells resistant to EGFR inhibition and found that this subpopulation of cells displayed properties of EMT, including an increased dependence on PDGFR signaling[38, 39]. During EMT, PDGFR signaling may largely supplant or supplement the role of EGFR signaling in promoting invadopodia formation in breast cancer cells. This also suggests that the EMT process may play roles in not only mediating local invasion and metastasis, but also providing an escape mechanism from growth factor inhibition.

### Src and its effectors

As the first proto-oncogene discovered, there is a large body of research focusing on not only the role of Src in cancer but also potential therapeutic interventions. It is generally recognized that Src plays multiple roles in carcinomas, promoting both proliferation and survival and driving invasion[40]. The essential role of Src activation in invadopodia formation suggests that Src inhibitors should effectively prevent invadopodia formation and ECM degradation in tumors. Several Src inhibitors are already in the clinic and used to treat chronic myelogenous leukemia by virtue of their ability to also inhibit Abl kinase[41]. Src activity is also upregulated in a wide variety of solid cancers, including colon, breast, gastric, and ovarian cancers[42]. Several pharmaceutical companies have therefore developed Src kinase inhibitors with varying levels of success. Most Src inhibitors that have progressed to clinical trials in solid tumors (Dasatinib, Bristol-Meyers Squibb; Saracatinib, AstraZeneca; and Bosutinib, Wyeth) work by competitively binding the ATP-binding site of Src[40]. Initial results from clinical trials of Src inhibitors in breast cancer have been mixed, with most single-agent trials resulting in no significant differences in survival or progression[43]. Combination therapeutics have resulted in more positive, although modest, effects [42].It is important to note, however, that all clinical trials regarding Src inhibitors in breast cancer have been conducted in unselected patient populations and the main readout for effectiveness has been tumor size and growth, not invasion. There are some indications, however, that patient stratification can predict responsiveness to Src kinase inhibition[44]. Novel Src inhibitors targeting the peptide binding site of Src rather than the ATP-binding site (KX2-391, Keryx Biopharmaceuticals) may also prove to be more effective in solid tumors, although clinical trials involving these compounds are still preliminary and underway[45].

Interestingly, a recent publication elucidated a detailed mechanisms for Src-kinase induced invadopodia formation[35]. Rather than directly phosphorylating cortactin, Src instead activates the Abl-related non-receptor tyrosine kinase Arg that is responsible for cortactin phosphorylation. In this system, cortactin tyrosine phosphorylation is transiently required for cortactin-mediated actin polymerization in invadopodia. This is particularly interesting, as Gleevec, a drug often used to target PDGFR signaling, also inhibits Arg activity[31]. The promiscuity of Gleevec could therefore target multiple levels of the signaling pathways regulating invadopodia formation, making it a promising target in selected patient populations[46].

### Metalloproteases

Several metalloproteases are enriched at invadopodia, including MMP2, MMP9, and MT1-MMP[47]. The transmembrane metalloprotease MT1-MMP is essential for invadopodia proteolytic activity: knockdown of MT1-MMP in multiple cell lines almost completely eliminates associated matrix degradation[19, 48]. In addition, recent work has also elucidated the vital role of MT-MMPs in mediating invasion through three-dimensional matrices[49].The central role of MT-MMPs in mediating extracellular proteolysis at invadopodia could be due to either its intrinsic collagenase/gelatinase activity or via activation of soluble MMPs by MT1-MMP[50]. There is also evidence that hydroxymate metalloprotease inhibitors prevent not only ECM proteolysis, but also invadopodia formation through an unknown mechanism[19].

As cancer cells must invade through both basement membranes and the ECM during metastasis, metalloproteases were quickly recognized as appealing targets to inhibit metastasis. Although results were promising in preclinical models, metalloprotease inhibitors have universally failed in clinical trials[51]. Once again, clinical trials with metalloprotease inhibitors to date have invariably used unselected patient populations, often with late-stage disease. Additionally, early metalloprotease inhibitors were broad-spectrum inhibitors of multiple metalloproteases and often had acute toxicities that severely limited therapeutic doses[51]. MMPs may also play anti-tumor functions in many tumors, as well. For example, MMP8-/- mice developed more papillomas upon carcinogen treatment[52].In recent years, there has been a reemergence of interest in more targeted inhibition of metalloproteases. In particular, the fully human monoclonal antibody DX-2400 (Dyax Corp.) that targets MT1-MMP, has shown great promise in preclinical models in inhibiting invasiveness of cancer cell lines[53]. In addition, a novel class of metalloprotease inhibitors, triple-helical transition state analogues, specifically targets the gelatinase and collagenase activities of metalloproteases (specifically MMP2 and 9)[54]. Clinically addressing the role of metalloproteases in breast cancer metastasis will involve not only designing trials to maximize the impact of the therapeutics, but also finding novel inhibitors with greater specificity and fewer negative side-effects.

## CONCLUSIONS

In order to directly target metastasis, essential regulators of the metastatic process must be identified. In addition, these targets should ideally be kinases or proteases with moieties amenable to chemical inhibition. Although EMT is beginning to be recognized as a key player in breast cancer, promising targets of inhibition regulating this process have been lacking. Our identification of PDGFRα and invadopodia as essential mediators of EMT-induced metastasis opens the door for clinical intervention of pathways regulating invadopodia function. These pathways include Src kinase, PDGFRα, and invadopodia-specific proteases. In order to test the hypothesis that inhibiting such pathways is effective, clinical trials most likely benefit from careful selection of patient populations based on our knowledge of invadopodia regulation. In addition, for both PDGFRα and metalloproteases, generation of more selective compounds may be necessary to realize positive clinical outcomes. Our study suggests that Twist1 and PDGFRα are effective predictors, not only of patient survival, but also for patient selection in clinical trials targeting invadopodia formation and function.

## References

[1] Jemal A, Tiwari RC, Murray T, Ghafoor A, Samuels A, Ward E, Feuer EJ, Thun MJ (2004). Cancer statistics, 2004. CA: A Cancer Journal for Clinicians.

[2] Ferlay J, Autier P, Boniol M, Heanue M, Colombet M, Boyle P (2007). Estimates of the cancer incidence and mortality in Europe in 2006. Ann Oncol.

[3] Fidler IJ (2003). The pathogenesis of cancer metastasis: the “seed and soil” hypothesis revisited. Nature Rev Cancer.

[4] Kalluri R, Weinberg RA (2009). The basics of epithelial-mesenchymal transition. J Clin Invest.

[5] Hay ED (1995). An overview of epithelio-mesenchymal transformation. Acta Anat (Basel).

[6] Peinado H, Olmeda D, Cano A (2007). Snail, Zeb and bHLH factors in tumour progression: an alliance against the epithelial phenotype?. Nature Rev Cancer.

[7] Yang J, Mani SA, Donaher JL, Ramaswamy S, Itzykson RA, Come C, Savagner P, Gitelman I, Richardson A, Weinberg RA (2004). Twist, a master regulator of morphogenesis, plays an essential role in tumor metastasis. Cell.

[8] Beslija S, Bonneterre J, Burstein H, Cocquyt V, Gnant M, Goodwin P, Heinemann V, Jassem J, Köstler WJ, Krainer M, Menard S, Petit T, Petruzelka L, Possinger K, Schmid P, Stadtmauer E (2007). Second consensus on medical treatment of metastatic breast cancer. Ann Oncol.

[9] Imming P, Sinning C, Meyer A (2006). Drugs, their targets and the nature and number of drug targets. Nature Rev Drug Discovery.

[10] Hopkins AL, Groom CR (2002). The druggable genome. Nature Rev Drug Discovery.

[11] Zlobec I, Lugli A Epithelial mesenchymal transition and tumor budding in aggressive colorectal cancer: Tumor budding as oncotarget. Oncotarget.

[12] Onder TT, Gupta PB, Mani SA, Yang J, Lander ES, Weinberg RA (2008). Loss of E-cadherin promotes metastasis via multiple downstream transcriptional pathways. Cancer Res.

[13] Tarone G, Cirillo D, Giancotti FG, Comoglio PM, Marchisio PC (1985). Rous sarcoma virus-transformed fibroblasts adhere primarily at discrete protrusions of the ventral membrane called podosomes. Exp Cell Res.

[14] Chen WT (1989). Proteolytic activity of specialized surface protrusions formed at rosette contact sites of transformed cells. J Exp Zool.

[15] Yamaguchi H, Oikawa T (2010). Membrane lipids in invadopodia and podosomes: Key structures for cancer invasion and metastasis. Oncotarget.

[16] Linder S (2009). Invadosomes at a glance. J Cell Sci.

[17] Linder S (2007). The matrix corroded: podosomes and invadopodia in extracellular matrix degradation. Trends Cell Biol.

[18] Seals DF, Azucena EF, Pass I, Tesfay L, Gordon R, Woodrow M, Resau JH, Courtneidge SA (2005). The adaptor protein Tks5/Fish is required for podosome formation and function, and for the protease-driven invasion of cancer cells. Cancer Cell.

[19] Ayala I, Baldassarre M, Giacchetti G, Caldieri G, Tetè S, Luini A, Buccione R (2008). Multiple regulatory inputs converge on cortactin to control invadopodia biogenesis and extracellular matrix degradation. J Cell Sci.

[20] Chen WT (1996). Proteases associated with invadopodia, and their role in degradation of extracellular matrix. Enzyme Prot.

[21] Buschman MD, Bromann PA, Cejudo-Martin P, Wen F, Pass I, Courtneidge SA (2009). The novel adaptor protein Tks4 (SH3PXD2B) is required for functional podosome formation. Mol Bio Cell.

[22] Eckert MA, Lwin TM, Chang AT, Kim J, Danis E, Ohno-Machado L, Yang J (2011). Twist1-induced invadopodia formation promotes tumor metastasis. Cancer Cell.

[23] Bowden ET, Onikoyi E, Slack R, Myoui A, Yoneda T, Yamada KM, Mueller SC (2006). Co-localization of cortactin and phosphotyrosine identifies active invadopodia in human breast cancer cells. Exp Cell Res.

[24] Yu J, Ustach C, Kim H-RC (2003). Platelet-derived growth factor signaling and human cancer. J Biochem Mol Biol.

[25] Hoch RV, Soriano P (2003). Roles of PDGF in animal development. Development.

[26] Li X, Kumar A, Zhang F, Lee C, Li Y, Tang Z, Arjuna P (2010). VEGF-independent angiogenic pathways induced by PDGF-C. Oncotarget.

[27] Kypta RM, Goldberg Y, Ulug ET, Courtneidge SA (1990). Association between the PDGF receptor and members of the src family of tyrosine kinases. Cell.

[28] Yang Y, Yuzawa S, Schlessinger J (2008). Contacts between membrane proximal regions of the PDGF receptor ectodomain are required for receptor activation but not for receptor dimerization. PNAS.

[29] Jechlinger M, Sommer A, Moriggl R, Seither P, Kraut N, Capodiecci P, Donovan M, Cordon-Cardo C, Beug H, Grünert S (2006). Autocrine PDGFR signaling promotes mammary cancer metastasis. J Clin Invest.

[30] Quintavalle M, Elia L, Condorelli G, Courtneidge SA (2010). MicroRNA control of podosome formation in vascular smooth muscle cells in vivo and in vitro. J Cell Biol.

[31] Manley PW, Cowan-Jacob SW, Buchdunger E, Fabbro D, Fendrich G, Furet P, Meyer T, Zimmermann J (2002). Imatinib: a selective tyrosine kinase inhibitor. Eur J Cancer.

[32] Cristofanilli M, Morandi P, Krishnamurthy S, Reuben JM, Lee B-N, Francis D, Booser DJ, Green MC, Arun BK, Pusztai L, Lopez A, Islam R, Valero V, Hortobagyi GN (2008). Imatinib mesylate (Gleevec) in advanced breast cancer-expressing C-Kit or PDGFR-beta: clinical activity and biological correlations. Ann Oncol.

[33] Mundhenke C, Strauss A, Schem C (2009). Significance of Tyrosine Kinase Inhibitors in the Treatment of Metastatic Breast Cancer. Breast Care.

[34] Gan HK, Seruga B, Knox JJ (2009). Sunitinib in solid tumors. Expert Opinion on Investigational Drugs.

[35] Mader CC, Oser M, Magalhaes MAO, Bravo-Cordero J, Condeelis JS, Koleske AJ, Gil-Henn H (2011). An EGFR-Src-Arg-cortactin pathway mediates functional maturation of invadopodia and breast cancer cell invasion. Cancer Res.

[36] Steger GG, Abrahámová J, Bacanu F, Brincat S, Brize A, Cesas A, Cufer T, Dank M, Duchnowska R, Eniu A, Jassem J, Kahán Z, Matos E, Padrik P, Plāte S, Pokker H (2010). Current standards in the treatment of metastatic breast cancer with focus on Lapatinib: a review by a Central European Consensus Panel. Wiener Klinische Wochenschrift.

[37] Lurje G, Lenz H-J (2009). EGFR signaling and drug discovery. Oncology.

[38] Thomson S, Petti F, Sujka-Kwok I, Epstein D, Haley JD (2008). Kinase switching in mesenchymal-like non-small cell lung cancer lines contributes to EGFR inhibitor resistance through pathway redundancy. Clin Exp Metastasis.

[39] Thomson S, Petti F, Sujka-Kwok I, Mercado P, Bean J, Monaghan M, Seymour SL, Argast GM, Epstein DM, Haley JD (2010). A systems view of epithelial-mesenchymal transition signaling states. Clin Exp Metastasis.

[40] Morgan L, Nicholson RI, Hiscox S (2008). SRC as a therapeutic target in breast cancer. Endocrine, Metabolic & Immune Disorders Drug Targets.

[41] Azam M, Nardi V, Shakespeare WC, Metcalf CA, Bohacek RS, Wang Y, Sundaramoorthi R, Sliz P, Veach DR, Bornmann WG, Clarkson B, Dalgarno DC, Sawyer TK, Daley GQ (2006). Activity of dual SRC-ABL inhibitors highlights the role of BCR/ABL kinase dynamics in drug resistance. PNAS.

[42] Mayer EL, Krop IE (2010). Advances in targeting SRC in the treatment of breast cancer and other solid malignancies. Clin Cancer Res.

[43] Kim LC, Song L, Haura EB (2009). Src kinases as therapeutic targets for cancer. Nature Rev Clin Oncol.

[44] Tryfonopoulos D, Walsh S, Collins DM, Flanagan L, Quinn C, Corkery B, McDermott EW, Evoy D, Pierce A, O'Donovan N, Crown J, Duffy MJ (2011). Src: a potential target for the treatment of triple-negative breast cancer. Ann Oncol.

[45] Rothschild SI, Gautschi O, Haura EB, Johnson FM (2010). Src inhibitors in lung cancer: current status and future directions. Clin Lung Cancer.

[46] Petrelli A, Giordano S (2008). From single- to multi-target drugs in cancer therapy: when aspecificity becomes an advantage. Curr Med Chem.

[47] Wolf K, Friedl P (2009). Mapping proteolytic cancer cell-extracellular matrix interfaces. Clin Exp Metastasis.

[48] Tatin F, Varon C, Génot E, Moreau V (2006). A signalling cascade involving PKC, Src and Cdc42 regulates podosome assembly in cultured endothelial cells in response to phorbol ester. J Cell Sci.

[49] Rowe RG, Li X-Y, Hu Y, Saunders TL, Virtanen I, Garcia de Herreros A, Becker K-F, Ingvarsen S, Engelholm LH, Bommer GT, Fearon ER, Weiss SJ (2009). Mesenchymal cells reactivate Snail1 expression to drive three-dimensional invasion programs. J Cell Biol.

[50] Hofmann UB, Westphal JR, Zendman AJ, Becker JC, Ruiter DJ, Muijen GN van (2000). Expression and activation of matrix metalloproteinase-2 (MMP-2) and its co-localization with membrane-type 1 matrix metalloproteinase (MT1-MMP) correlate with melanoma progression. J Pathol.

[51] Gialeli C, Theocharis AD, Karamanos NK (2011). Roles of matrix metalloproteinases in cancer progression and their pharmacological targeting. FEBS J.

[52] Balbín M, Fueyo A, Tester AM, Pendás AM, Pitiot AS, Astudillo A, Overall CM, Shapiro SD, López-Otín C (2003). Loss of collagenase-2 confers increased skin tumor susceptibility to male mice. Nat Genet.

[53] Devy L, Huang L, Naa L, Yanamandra N, Pieters H, Frans N, Chang E, Tao Q, Vanhove M, Lejeune A, Gool R van, Sexton DJ, Kuang G, Rank D, Hogan S, Pazmany C (2009). Selective inhibition of matrix metalloproteinase-14 blocks tumor growth, invasion, and angiogenesis. Cancer Res.

[54] Lauer-Fields J, Brew K, Whitehead JK, Li S, Hammer RP, Fields GB (2007). Triple-helical transition state analogues: a new class of selective matrix metalloproteinase inhibitors. J Am Chem Soc.

